# Insights From Amniotic and Umbilical Cord Mesenchymal Stem Cells in Wound Healing

**DOI:** 10.1111/jcmm.70679

**Published:** 2025-06-19

**Authors:** Nong‐er Shen, Yue Wu, Kaichuang Yang, Xiuling Xv, Gang Lu, Ruolang Pan, Yang Jin

**Affiliations:** ^1^ Department of Pharmacy, Center for Clinical Pharmacy, Cancer Center Zhejiang Provincial People's Hospital (Affiliated People's Hospital, Hangzhou Medical College) Hangzhou Zhejiang China; ^2^ Department of Neurosurgery, Center for Rehabilitation Medicine Zhejiang Provincial People's Hospital (Affiliated People's Hospital, Hangzhou Medical College) Hangzhou Zhejiang China; ^3^ Department of Gynecology and Obstetrics, Hangzhou Hospital of Traditional Chinese Medicine Affiliated Hangzhou TCM Hospital of Zhejiang Chinese Medical University Hangzhou China; ^4^ Key Laboratory of Cell‐Based Drug and Applied Technology Development in Zhejiang Province Institute for Cell‐Based Drug Development of Zhejiang Province Hangzhou China

**Keywords:** amniotic membrane, inflammatory microenvironment, mesenchymal stem cell, skin repair, umbilical cord

## Abstract

Skin repair is a complex physiological process that involves the coordinated actions of various cell types. This study examines the distinct roles of amniotic mesenchymal stem cells (A‐MSCs) and umbilical cord mesenchymal stem cells (UC‐MSCs) in skin healing using a mouse model. Gene Ontology (GO) and Kyoto Encyclopedia of Genes and Genomes (KEGG) pathway analyses revealed significant differences in gene expression between A‐MSCs and UC‐MSCs. Specifically, A‐MSCs exhibited upregulation of genes associated with extracellular matrix (ECM) organisation and cell migration, thereby enhancing their tissue remodelling capabilities. In contrast, UC‐MSCs demonstrate increased expression of genes involved in angiogenesis and anti‐inflammatory responses, highlighting their role in creating a favourable healing environment. These findings highlight the unique therapeutic potentials of A‐MSCs and UC‐MSCs in skin repair strategies. Although MSCs hold promise in regenerative medicine, challenges such as optimal cell selection and modulation of the inflammatory microenvironment remain to be addressed. Our research emphasises the need for continued research related to properties of MSCs to refine therapeutic approaches for effective wound healing.

## 1 Introduction

Acute trauma refers to a short‐term injury that typically heals within a few days to a week, such as surgical incisions. The process of wound repair is a highly coordinated and precisely regulated biological event that involves various cells, cytokines and signalling pathways [1, 2, 3]. This topic holds particularly significance in treatment of burns, plastic surgery and tissue repair and reconstruction. Traditional approaches for managing skin injuries primarily encompass dressings, negative pressure wound therapy, autologous skin grafting and high‐pressure wound therapy [4, 5]. However, these methods have limitations, including high costs, delays in removal of necrotic tissues, need for surgical debridement and risks related to oxygen toxicity. Despite advancements in surgical techniques and repair materials over the years, achieving ‘perfect’ wound healing—defined as complete tissue regeneration—remains challenging. Such ‘Imperfect’ repairs processes can adversely impact a patient's appearance, functionality, and overall quality of life while also resulting in significant waste of medical resources and imposing substantial societal burdens. A key focus of research on wound repair is to understand the mechanisms underlying stimulation of cell proliferation and differentiation to enable the formation of functional tissues and ensuring precise arrangement of various cell types during the healing process. Consequently, the medical community continues to pursue methods that could lead to ‘perfect’ wound healing.

Initial response of the body to a wound involves blood vessel constriction and platelet activation, leading to the formation of a fibrin clot that arrests bleeding and provides a scaffold for inflammatory cells [6, 7]. Neutrophils infiltrate this clot, serving as the first line of defence against infection, while monocytes proliferate and differentiate into macrophages approximately 48 h post‐injury, facilitating tissue activation [8, 9]. As the inflammatory phase ends, the healing process transitions to the proliferative phase, wherein a granulation tissue is formed along with re‐epithelialisation and angiogenesis. Fibroblasts play a crucial role by contracting the wound and supporting ECM formation at this stage. Endothelial cells proliferate to create new blood vessels, supported by activated basal layer cells [10]. Although wound closure is often seen as the end of healing, remodelling can continue for months or years, thereby determining scarring outcomes. During this phase, type III collagen in granulation tissue is gradually replaced by the stronger type I collagen via a process involving matrix metalloproteinases (MMPs) and their inhibitors, tissue inhibitors of metalloproteinases (TIMPs). An imbalance between TIMPs and MMPs can lead to abnormal remodelling of ECM or chronic wounds [11, 12, 13]. Myofibroblasts undergo apoptosis upon completion of healing; however, if they fail to do so and engage in excessive remodelling, hypertrophic scars may form [14, 15]. Macrophages are vital in this remodelling process and exhibit a fibrinolytic phenotype that helps degrade excess ECM and clear debris [16, 17]. Overall, these processes highlight the complexity of wound healing and the intricate balance required for optimal recovery.

With rapid progress in tissue engineering, cell therapy is gaining increasing attention across multiple disciplines. Research indicates that stem cells play an essential role in regenerative medicine and are crucial for effective wound repair [18, 19, 20]. Among these, mesenchymal stem cells (MSCs) are the most frequently utilised seed cells because of their self‐renewal capabilities and potential for multidirectional differentiation. MSC transplantation is generally well‐tolerated with minimal risk of rejection. These cells contribute to injury repair through intercellular communication and the secretion of bioactive factors, thereby enhancing the efficiency of wound healing. Furthermore, MSCs exhibit higher expression levels of collagen, fibroblast growth factor (FGF) and vascular endothelial growth factor (VEGF) compared to natural dermal cells, significantly boosting healing rates [21, 22]. Currently, clinical trials investigating the use of MSCs for treating various skin injuries are a prominent area of research, with preliminary evidence supporting their effectiveness and safety in promoting wound regeneration. However, the efficacy of these treatments can vary based on the source of MSCs used. Therefore, this study aims to compare the effects of umbilical cord‐derived mesenchymal stem cells (UC‐MSCs) and amniotic membrane‐derived mesenchymal stem cells (A‐MSCs) on skin injury repair while carrying out an initial analysis on their underlying mechanisms.

## 2 Materials and Methods

### 2.1 UC‐MSCs and A‐MSCs Isolation and Culture

Human UC‐MSCs and A‐MSCs were provided by S‐Evans Biosciences (Hangzhou, China). The collection of human samples was approved by the Ethics Committee of S‐Evans Biosciences (No. 2020‐01). For UC‐MSCs, umbilical cords were collected from healthy donors within 4 h of delivery. The cords were rinsed with 75% ethanol for disinfection and then cut into small segments. Wharton's jelly was carefully extracted using blunt dissection, followed by enzymatic digestion with collagenase type I (Sigma‐Aldrich; SCR103) to release the MSCs. The resulting cell suspension was filtered, centrifuged and cultured in MSC medium (alpha modification of Eagle's medium, Gibco; 32561037) supplemented with 10% FBS (Gibco; A5256701), 100 U/mL penicillin and 100 μg/mL streptomycin (Sangon Biotech; E607011). For A‐MSCs, amniotic membranes were obtained from the placenta after delivery. The membranes were mechanically peeled from the underlying chorion and subjected to enzymatic digestion using trypsin and collagenase to isolate the cells, and then cultured in MSC medium. Both cells were incubated at 37°C in 5% CO_2_ using a humidified incubator, and passaged by 0.25% trypsin–EDTA (Gibco; 25200072) digestion at a 1:4 ratio.

### 2.2 Characterisation of UC‐MSCs and A‐MSCs and Flow Cytometry Analysis

Both UC‐MSCs and A‐MSCs were characterised by flow cytometry to confirm their mesenchymal stem cell identity and multilineage differentiation potential. Specifically, the cells were evaluated for morphology and surface marker expression. To further assess their differentiation capabilities, in vitro osteogenic, adipogenic and chondrogenic inductions were conducted according to established protocols. Post‐differentiation, mineralisation was quantified using Alizarin Red S staining, lipid accumulation was visualised with Oil Red O staining, and proteoglycan deposition was confirmed through Alcian blue staining. Additionally, phenotypic characteristics of UC‐MSCs and A‐MSCs at passage 3 were analysed by flow cytometry using antibodies against CD14 (BD Biosciences; 555397), CD34 (BD Biosciences; 555822), CD73 (BioLegend; 344004), CD105 (BioGems; 17111‐60), HLA‐DR (BD Biosciences; 555811) and CD90 (BioLegend; 328110), with isotype‐matched antibodies (PE, BD Biosciences; 555749; FITC, BD Biosciences; 555573) serving as a negative control.

### 2.3 Proliferation Assay

For the Cell Counting Kit‐8 (CCK‐8) assay, cells were seeded into 96‐well plates at a density of 2000 cells per well in 100 μL of complete medium. At 0, 24, 48 and 72 h post‐seeding, 10 μL of CCK‐8 solution (Beyotime; C0037) was added to each well. Absorbance was measured at 450 nm using a microplate reader at each time point, with three replicates for each measurement.

### 2.4 In Vitro Migration Assay

Migratory capabilities of cells were assessed using Transwell chambers (Corning; CLS3428). A total of 5 × 10^4^ cells were seeded in the upper chamber, while 800 μL of medium containing 10% fetal bovine serum (FBS) was added to the lower chamber. Following a 24‐h incubation period, the number of cells that migrated to the lower surface was quantified. Data were derived from three independent experiments.

### 2.5 Animal Studies

Twenty‐four healthy Kunming ICR mice (aged 10–12 weeks and weighing 23–30 g) were bought from the Shanghai SLAC Laboratory Animal Co. Ltd. [Certificate number: SCXK (Shanghai) 2017‐0005] and maintained in the SPF laboratory of a local animal facility. The experimental protocol was approved by the local medical animal experiment ethics committee. To establish a mouse skin injury model, anaesthesia was administered via intraperitoneal injection of 10% chloral hydrate at a dosage of 300 mg/kg. The dorsal area was shaved, and a full‐thickness circular wounds (10 mm in diameter) were created using aseptic surgical scissors, ensuring that the underlying fascia remained intact. The mice were randomly divided into three treatment groups and housed individually to monitor healing over time. The experimental group received subcutaneous injections of 0.5 million MSCs suspended in 50 μL of PBS at three sites along the wound edge: 12 o'clock, 4 o'clock and 8 o'clock positions, each 4–5 mm from the wound margin. The mode of administration of MSCs as well as the injection dose were selected based on previous studies demonstrating optimal efficacy in a mouse wound model [23, 24]. The control group was injected with an equal volume of PBS at the same locations. Wound healing was assessed through macroscopic observation and measurement of wound area at designated intervals.

### 2.6 Immunohistochemistry (IHC) and Confocal Microscopy

For the histological analysis of mouse skin tissue, haematoxylin and eosin (H&E) and Masson's trichrome staining were performed. Skin samples were first fixed in 4% formalin at 4°C for 8 h, followed by dehydration through a series of ethanol solutions (70%, 95% and absolute ethanol). Next, the samples were immersed in xylene and embedded in paraffin. Sections of 4 μm thickness were cut and stained with H&E, and later they were stained with Harris' haematoxylin for nuclear visualisation, rinsed and counterstained with eosin to highlight cytoplasmic structures. For Masson's Trichrome staining, the deparaffinised sections were sequentially stained with Weigert's iron haematoxylin, Biebrich scarlet‐acid fuchsin and Aniline blue. This method allowed for differentiation of collagen (blue), muscle fibres (red) and nuclei (dark brown/black), thus providing a comprehensive view of the tissue architecture. The stained sections were observed under a light microscope to assess histopathological changes and collagen distribution in the skin.

### 2.7 Enzyme‐Linked Immunosorbent Assays (ELISAs) for Tumour Necrosis Factor‐Alpha (TNF‐α) and Transforming Growth Factor‐Beta (TGF‐β)

To quantify the levels of TNF‐α and TGF‐β, we employed ELISAs using commercially available kits (mlbio, ml002095; mlbio, ml057830). For TNF‐α, a sandwich ELISA method was utilised, where samples were added to 96‐well plates pre‐coated with monoclonal antibodies specific to human TNF‐α. Following incubation, a biotinylated detection antibody was added, which formed a complex with the captured TNF‐α. After washing, an avidin‐biotin‐peroxidase complex was added, followed by the addition of the substrate tetramethylbenzidine (TMB), which produced a colorimetric reaction that was measured at 450 nm. The concentration of TNF‐α was determined based on a standard curve. Similarly, TGF‐β levels were measured using a solid‐phase sandwich ELISA.

### 2.8 RNA Extraction and Sequencing

Total RNA was extracted using the Total Exosome RNA and Protein Isolation Kit (Invitrogen; 4478545) according to the manufacturer's protocol, and the amount was quantified using a NanoDrop spectrophotometer. The obtained RNA was delivered to Lianchuan Biotechnology Company for library construction and subsequent RNA sequencing (RNA qualification standards are: OD260/280 = 1.18–2.2, OD260/230 ≥ 2.0, RIN ≥ 6.5, 28S:18S ≥ 1.0). For RNA sequencing (RNA‐Seq), biological replicates were sequenced with *n* = 3 per group. Batch effects were assessed and minimised to ensure data quality. RNA‐Seq was performed using the Illumina HiSeq 4000 sequencing system. The MapSplice program was used for RNA‐Seq data mapping. Transcripts per kilobase per million was used to measure the expression levels of each gene. The number of genes with differential expression up‐regulated (log_2_FC ≥ 2 and *q* < 0.05) and down‐regulated (log_2_FC ≤ −2 and *q* < 0.05) in each comparison group was counted. The Lianchuan Cloud tool platform was used to perform GO and KEGG enrichment on the differentially expressed genes in each group. The RNA‐Seq data was deposited in the BioProject under accession number PRJNA1270162.

### 2.9 Statistical Analysis

Data are expressed as means ± standard deviations (SD) from at least three independent experiments. Student's *t*‐test was employed to assess differences between two groups when appropriate. For multiple group comparisons, one‐way analysis of variance (ANOVA) followed by Tukey's post hoc test was performed. A *p*‐value of < 0.05 was considered statistically significant.

## 3 Results

### 3.1 Isolation and Characterisation of UC‐MSCs and A‐MSCs

**FIGURE 1:**
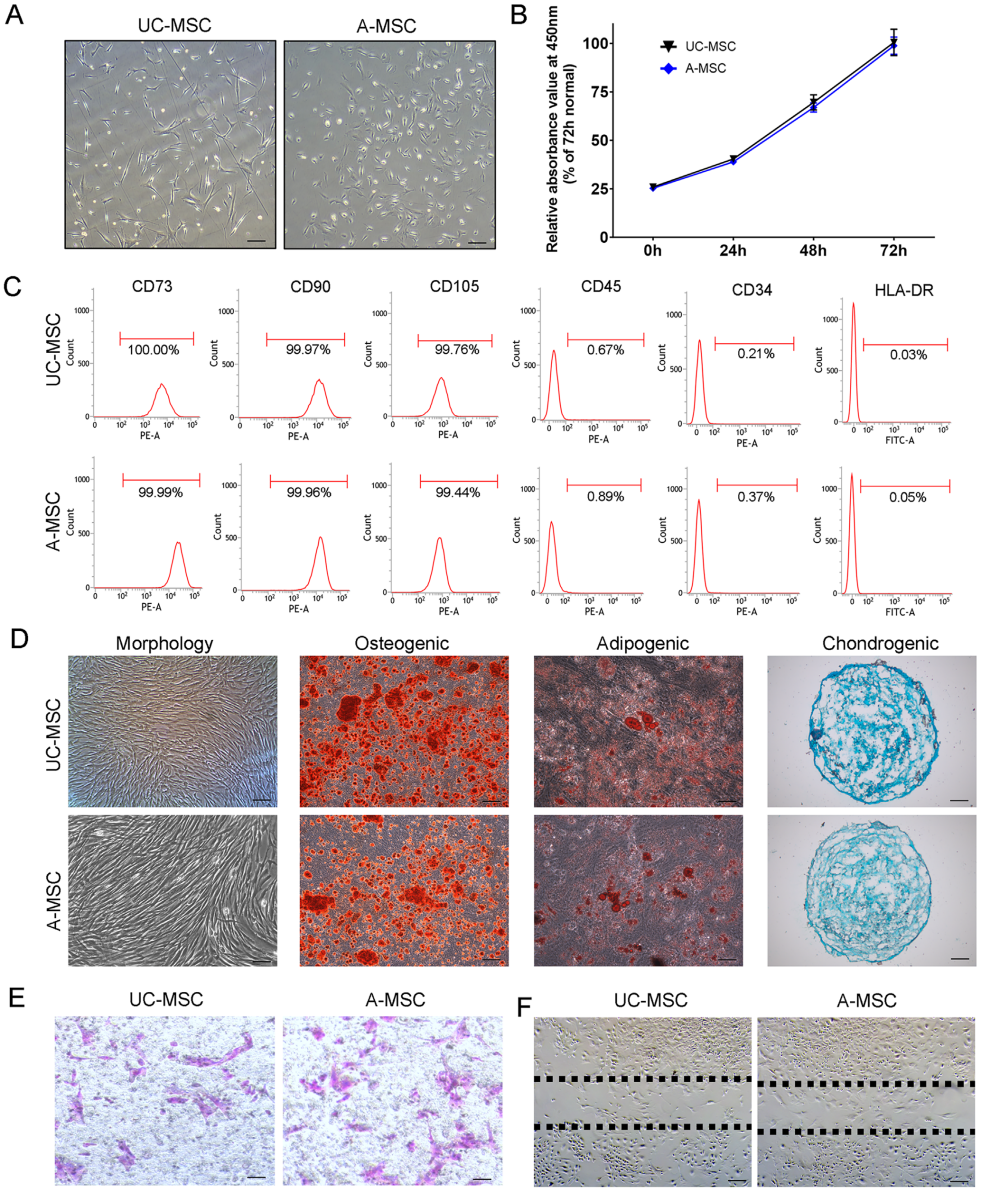
Isolation and characterisation of UC‐MSCs and A‐MSCs. (A) Both UC‐MSCs and A‐MSCs exhibit a typical spindle‐shaped morphology. Scale bar = 100 μm. (B) The proliferation of the two cells was evaluated by CCK8 assay. (C) These cells are positively stained for the common MSC‐associated markers, CD73, CD90, CD105, and have no expression of haematopoietic markers CD45, CD34 and HLA‐DR. (D) Following the culture in a specific differentiation medium for 2–3 weeks, the isolated UC‐MSCs and A‐MSCs successfully differentiated into mesenchymal derivatives, including osteoblasts (Alizarin Red labeling), adipocytes (Oil Red O labeling) and chondrocytes (Alcian blue). Scale bar = 50 μm. (E) The transwell was adopted to evaluate the migration ability of the two types of cells. Scale bar = 50 μm. (F) The wound healing assays were further adopted for the migration ability of the cells. Scale bar = 100 μm.

Both UC‐MSCs and A‐MSCs were successfully isolated and characterised according to established criteria for mesenchymal stem cells. Both cell types exhibited adherent growth in plastic culture dishes and displayed a fibroblast‐like morphology (Figure 1A). CCK8 experiments showed that the proliferation rates of both types of cells were similar (Figure 1B). Flow cytometry analysis confirmed that both UC‐MSCs and A‐MSCs expressed the requisite surface markers, specifically CD45−, CD11b−, CD19−, CD44+, CD73+, CD90+ and CD105+ (Figure 1C), thus meeting the minimum requirements for MSC classification, as recommended by the International Society for Cellular Therapy (ISCT) guidelines in 2006. Furthermore, under appropriate induction conditions, both UC‐MSCs and A‐MSCs demonstrated the ability to differentiate into osteoblasts, adipocytes and chondrocytes, confirming their multi‐lineage differentiation potential (Figure 1D). These findings establish that both UC‐MSCs and A‐MSCs possess fundamental characteristics that are necessary for their application in regenerative medicine. On this basis, we further investigated the migration and mobilisation ability of the two types of cells; results of the transwell migration assay (Figure 1E) as well as the wound healing assay (Figure 1F) demonstrated that the migration ability of the two cell types was basically similar, with no statistical difference.

### 3.2 Local Injection of MSCs Improved the Repair of Skin Injuries in Mice

**FIGURE 2:**
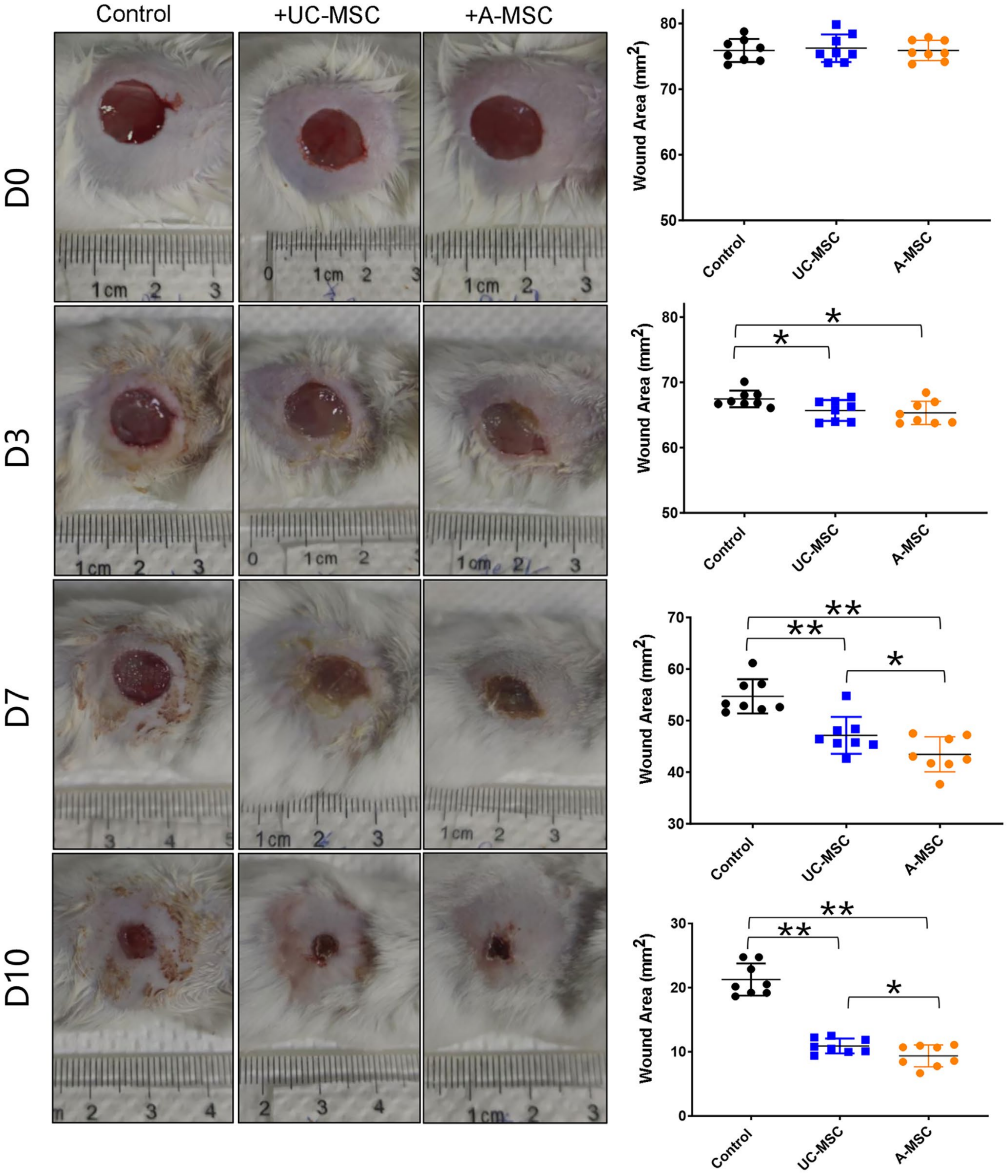
Local injection of MSCs improved the repair of skin injuries in mice. Representative photos of mice in each group, time points include 0, 3, 7 and 10 days after surgery. The wound area at each time point was calculated by imageJ software and compared between groups. *n* = 8, *p < 0.05, **p < 0.01.

As shown in Figure 2, size of the original wound on the back of the mouse was 1.0 cm in diameter. Over time, the wound area decreased in all groups, with the rate of healing in the UC‐MSC and A‐MSC treatment groups being significantly higher than that in PBS control group. Three days post‐surgery, scabs formed on the wounds of mice in the cell therapy groups, appeared light yellow, whereas redness and swelling around the wounds in the PBS control group were more pronounced than in experimental groups. The wound areas in the cell therapy groups were significantly smaller than in the control group. By day 7 post‐surgery, inflammatory response in all the groups showed significant improvement; scabs were dry and wound areas were further reduced. The wound area observed in the control group measured 39.06 ± 0.90 mm^2^, while that in UC‐MSC and A‐MSC treatment groups measured 31.51 ± 0.50 and 27.5 ± 0.44 mm^2^, respectively, indicating a better healing outcome for A‐MSC group than for UC‐MSC group (*p* < 0.05). On day10 post‐surgery, measurements of wound area in the control group was 24.98 ± 0.52 and 15.26 ± 0.36 mm^2^ in the UC‐MSC group and 10.96 ± 0.38 mm^2^ in A‐MSC group, once again demonstrating statistically significant differences among the groups (*p* < 0.05). Ultimately, complete wound closure occurred on days 17, 13 and 12 in the PBS control, UC‐MSC treatment and A‐MSC treatment groups, respectively, with beginning of hair growth around the wound edges in both the MSC treatment groups.

### 3.3 Histological Analysis of Skin Repair on Day 10 Post‐Surgery

**FIGURE 3:**
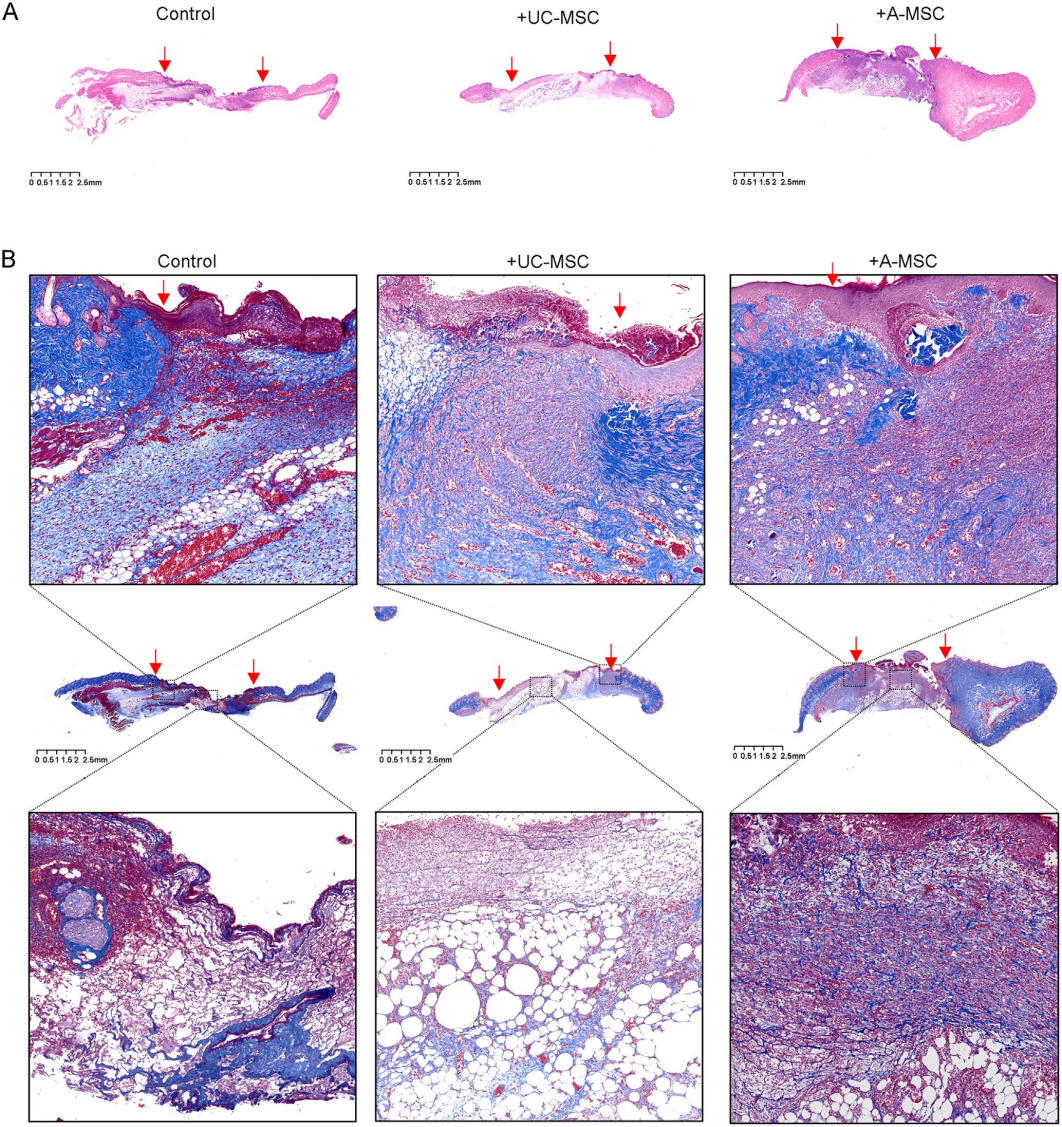
Histological analysis of skin repair on day 10 post‐surgery. (A) Haematoxylin and eosin (H&E) staining reveals significantly improvement of the wound tissue in MSC treatment groups compared to the control group. The A‐MSC treatment group shows tightly arranged cells with greater thickness than both the PBS and UC‐MSC treatment groups, indicating superior cell proliferation effects in the newly formed skin. (B) Masson's staining illustrates newly generated cavity‐like or cord‐like blood vessels at the edges of wounds across all groups; however, these structures are less abundant in the control group, significantly increased in the A‐MSC treatment group, and most pronounced in the UC‐MSC treatment group. The lower panel shows collagen fibre structures within the wounds: Loose in the control group while more neatly arranged in both MSC treatment groups, with denser collagen deposition observed in the A‐MSC treatment group. Colorimetric analysis indicates greater collagen accumulation and more effective collagen fibre repair in the A‐MSC group. Both MSC‐treated groups also show increased adipocyte production at the wound site, particularly evident in the UC‐MSC‐treated group. The arrows indicate the location of the wound.

As shown in Figure 3A, we compared the differences in skin repair among the groups of mice on the 10th day after surgery. H&E staining revealed that inflammatory cell infiltration in the wound tissue of the PBS control group was significantly higher than in the two MSC treatment groups, which exhibited relatively less inflammatory infiltration. The cells in the wound area of the A‐MSC treatment group were tightly arranged and demonstrated a significantly greater thickness than those in both the PBS control group and UC‐MSC treatment group, indicating superior effects on cell proliferation in the newly formed skin, although the scabs were more prominent. Masson's staining, illustrated in Figure 3B (upper panel), showed newly generated cavity or cord‐like blood vessels at the edges of wounds across all the groups. However, these were less abundant in the PBS group, significantly increased in the A‐MSC treatment group, and most pronounced in the UC‐MSC treatment group. As shown in the lower panel of Figure 3B, collagen fibre structures within the wounds were observed to be loose in the PBS group, whereas those in both the MSC treatment groups were more neatly arranged, with the A‐MSC treatment group exhibiting denser collagen deposition. Colorimetric analysis of blue collagen deposition indicated that the A‐MSC‐treated group exhibited greater collagen accumulation and more effective collagen fibre repair. Interestingly, both the MSC‐treated groups showed increased adipocyte production at the wound site, which was particularly evident in the UC‐MSC‐treated group. Collectively, these results suggest that MSC treatment enhances epidermal repair and contributes positively to skin damage recovery through improved collagen deposition and cell proliferation.

### 3.4 Determination of Serum TNF‐α and TGF‐β Levels in Wound Tissue

**FIGURE 4:**
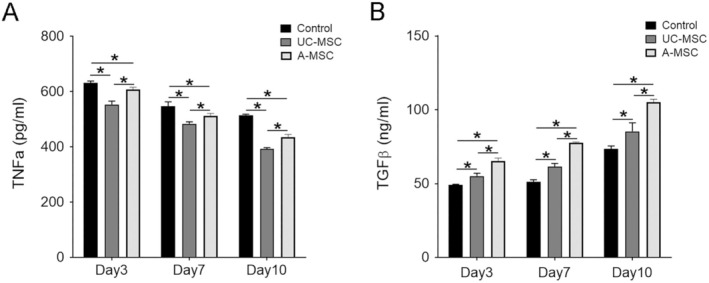
Determination of serum TNF‐α and TGF‐β levels. (A, B) The expression trends and significance of TNF‐α and TGF‐β were assessed over time post‐surgery. *p < 0.05.

In the process of skin damage repair, the trend of expression and significance of TNF‐α and TGF‐β has important biological and clinical significance [25, 26]. Therefore, we collected samples at different time points for testing. As shown in Figure 4, at 3 days post‐surgery, serum TNF‐α level in the control group was 631.55 ± 7.04 pg/mL. In comparison, the serum TNF‐α levels in the A‐MSC treatment group significantly decreased to 608.23 ± 7.74 pg/mL (*p* < 0.05), while those in the UC‐MSC treatment group were further reduced to 553.69 ± 12.2 pg/mL (*p* < 0.05). Overall comparisons among the groups indicated statistically significant differences (*p* < 0.05). By day 7, serum TNF‐α levels were 546.85 ± 16.43 pg/mL for the control group, 513.45 ± 7.99 pg/mL for the A‐MSC group (*p* < 0.05) and 484.15 ± 6.62 pg/mL for the UC‐MSC group (*p* < 0.05), reflecting continued reductions in inflammatory markers with MSC treatment. On day10, TNF‐α levels further decreased to 514.86 ± 3.35 pg/mL in the control group, 436.32 ± 8.74 pg/mL in the A‐MSC group and 392.96 ± 4.80 pg/mL in the UC‐MSC group, with significant differences noted across the groups. For TGF‐β, 3 days post‐surgery, serum levels were recorded at 49.27 ± 0.29 ng/mL in the control group, with significant increases observed in both MSC treatment groups: A‐MSC at 65.42 ± 1.90 ng/mL (*p* < 0.05) and UC‐MSC at 55.14 ± 1.84 ng/mL (*p* < 0.05). By day 7, TGF‐β levels were 51.26 ± 1.37 ng/mL for controls, 77.81 ± 1.34 ng/mL for A‐MSCs (*p* < 0.05) and 61.58 ± 2.07 ng/mL for UC‐MSCs (*p* < 0.05). On day10, TGF‐β levels continued to rise to 73.58 ± 1.92 ng/mL in the control group, while those in A‐MSCs reached 85.40 ± 5.79 ng/mL and those in UC‐MSCs peaked at 105.30 ± 1.77 ng/mL, with statistically significant differences observed across all the groups.

### 3.5 Transcriptomic Profiles and Differential Expression Analysis

**FIGURE 5:**
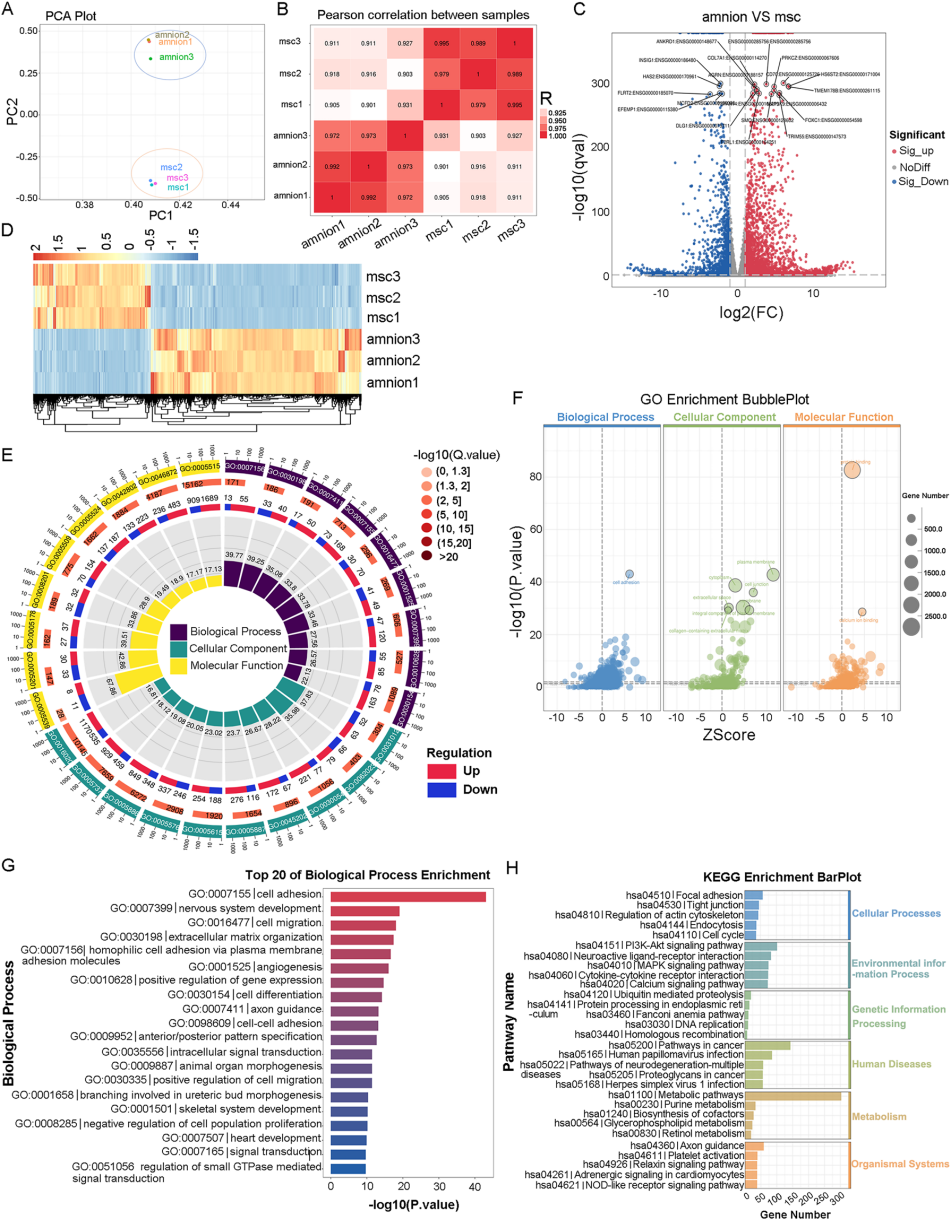
Transcriptomic profiles and differential expression analysis. (A) Principal component analysis (PCA) illustrates distinct clustering of treatment groups into two populations. (B) The Pearson correlation heatmap indicates strong correlations among samples within each group. (C) Volcano plots visualise differential gene expression between UC‐MSCs and A‐MSCs, the top 20 genes with significant differences between groups were labelled based on their *p*‐values. (D) A heatmap displays the top 100 transcripts. (E) The outermost circle displays the top enriched GO terms (ranked by smallest *p*‐ or *q*‐values), with an external scale indicating gene counts. Different colours represent the three main GO categories. The second circle shows the number of genes annotated to each GO term, with colour intensity reflecting the −log_10_(*p*‐value) or −log_10_(*q*‐value). The third circle provides a breakdown of upregulated and downregulated genes for each GO term, with red representing upregulation and blue representing downregulation. The innermost circle indicates the percentage of enrichment factor (Rich.Factor). (F) GO enrichment analysis shows top enriched GO terms ranked by smallest *p*‐values, with bubble charts highlighting gene distributions across GO terms. (G, H) Significant enrichments were noted in biological processes such as cell adhesion, migration, ECM organisation and angiogenesis. amnion, A‐MSC group; msc, UC‐MSC group.

Given the distinct effects of UC‐MSCs and A‐MSCs on skin damage repair, we analysed their gene expression profiles using RNA‐Seq. Principal component analysis (PCA) revealed that the treatment groups clustered into two distinct cell populations (Figure 5A). In the Pearson correlation heatmap, both the horizontal and vertical axes represent individual samples, with colour intensity indicating the degree of correlation between the samples. A deeper red hue (correlation coefficient closer to 1) indicates stronger correlation, whereas lighter colours suggest weaker relationships, confirming high concordance among samples within the same group (Figure 5B). Using R packages such as ggplot2 and ggrepel, we visualised differential gene expression between the two groups using volcano plots (Figure 5C) and a heatmap of the top 100 transcripts (Figure 5D). Differentially expressed genes were identified using thresholds of |log_2_FC| ≥ 1 and *q* < 0.05, revealing 1693 significantly upregulated genes and 3037 significantly downregulated genes in UC‐MSCs compared to those in A‐MSCs.

Gene Ontology (GO), an international standardised system for categorising gene functions, was used to further explore these differences. As shown in Figure 5E, the GO enrichment circle plot illustrates the top enriched GO terms ranked by the smallest *p*‐ or *q*‐values, with an external scale indicating gene counts. Additionally, we used Goplot to create bubble charts highlighting the distribution of upregulated and downregulated genes across the GO terms (Figure 5F). In these charts, the *y*‐axis represents −log_10_(*p*‐value) for GO enrichment analysis, whereas the *x*‐axis represents *Z*‐scores, indicating the extent of gene expression differences. Grey lines indicate *p*‐value thresholds of 0.01 and 0.05. Bubble size corresponds to the number of significantly differentially expressed genes within each GO term, whereas bubble colour denotes the GO category. The analysis revealed that the differentially expressed genes were significantly enriched in biological processes such as cell adhesion (GO:0007155), cell migration (GO:0016477), extracellular matrix organisation (GO:0030198) and angiogenesis (GO:0001525). KEGG pathway analysis further indicated significant enrichment in pathways related to focal adhesion, tight junctions, regulation of actin cytoskeleton, endocytosis and cell cycle.

**FIGURE 6:**
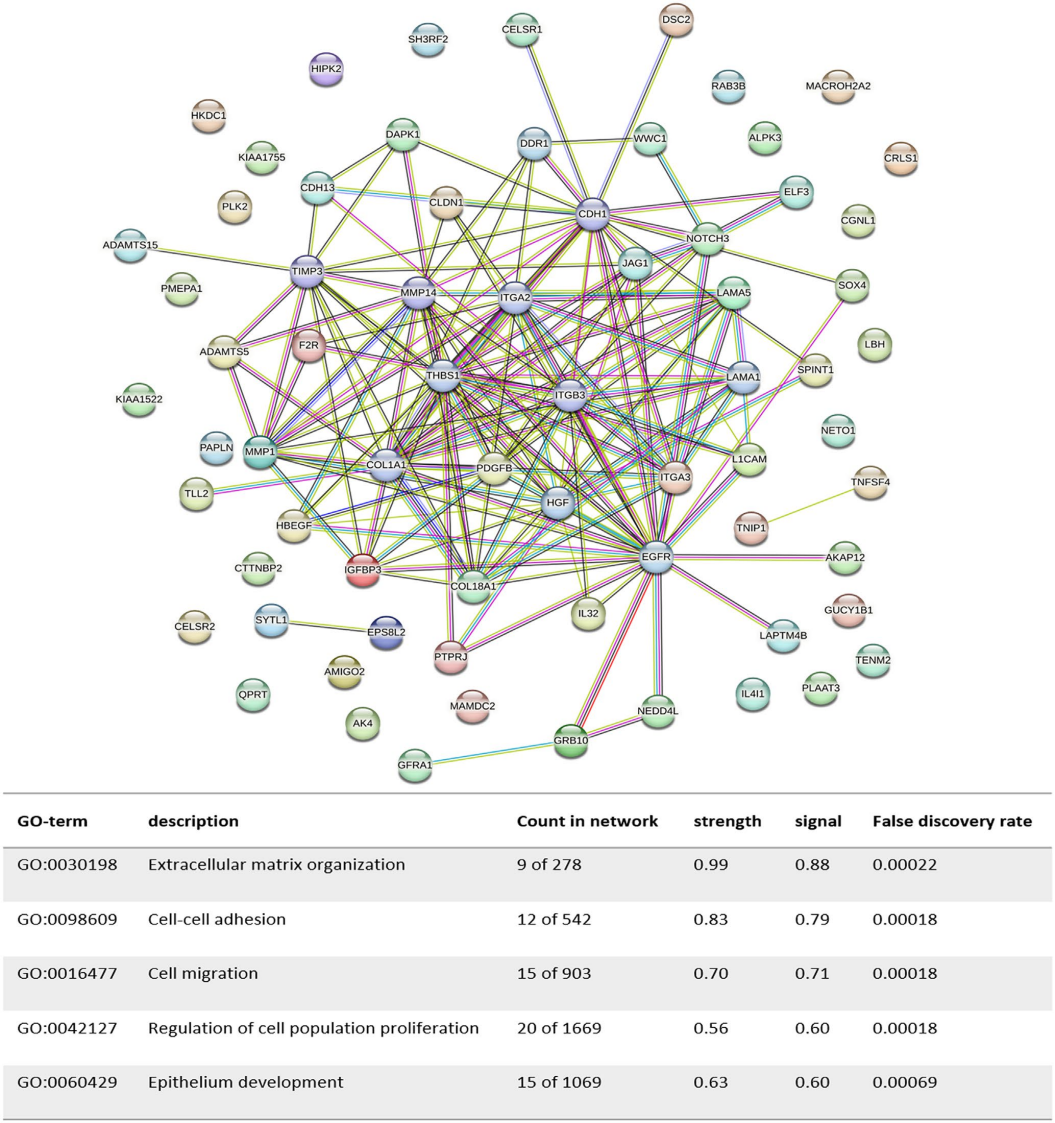
Protein–protein interaction analysis of differentially expressed genes. The biological processes analysis indicates that genes highly expressed in A‐MSCs are significantly enriched in GO terms related to extracellular matrix organisation, cell–cell adhesion, cell migration, regulation of cell population proliferation and epithelium development.

**FIGURE 7:**
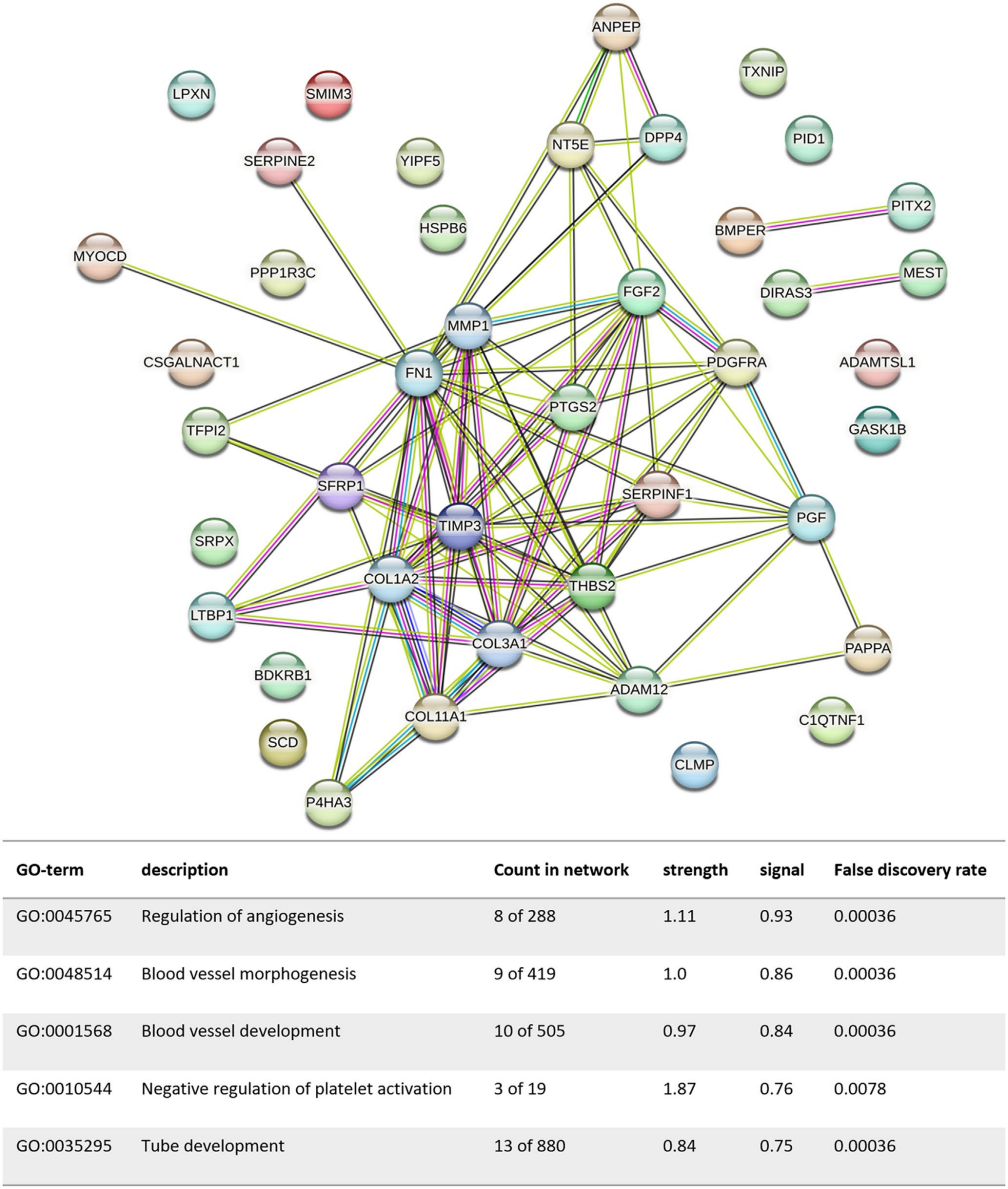
Protein–protein interaction analysis of differentially expressed genes. The biological processes analysis indicates that genes elevated in UC‐MSCs are enriched in terms associated with angiogenesis regulation, blood vessel morphogenesis, blood vessel development, negative regulation of platelet activation and tube development.

To further analyse molecular relationships among the implicated genes and proteins, we performed protein–protein interaction (PPI) analysis utilising the STRING database. Both the gene sets were subjected to GO enrichment analysis. As illustrated in Figure 6, biological processes analysis revealed that genes highly expressed in A‐MSCs were significantly enriched in GO terms such as extracellular matrix organisation, cell–cell adhesion, cell migration, regulation of cell population proliferation and epithelium development. In contrast, genes with elevated expression in UC‐MSCs were enriched in terms of regulation of angiogenesis, blood vessel morphogenesis, blood vessel development, negative regulation of platelet activation and tube development (Figure 7). These findings align with our earlier observations of differential effects between the two cell types during the wound healing process in mouse skin.

## 4 Discussion

Skin repair is one of the most complex processes in human physiology, requiring the interplay of various cell types. The epidermis serves as the outermost waterproof barrier, which protects the body from harsh environmental conditions. Within this layer, the sebaceous glands, sweat glands and hair follicles are essential for maintaining skin health. The extracellular matrix (ECM) is widely distributed throughout the dermis, providing support for blood vessels and mechanoreceptors that bolster skin immunity, deliver vital nutrients, and enhance skin strength and resilience [27, 28, 29]. Beneath the dermis lies a substantial layer of subcutaneous adipose tissue, which acts as an energy reservoir and supplies crucial growth factors and immune cells necessary for dermal development. These immune cells continuously monitor skin health and assess damage to skin [30, 31]. In the event of injury, a coordinated response among various cell types across these three layers initiates processes such as haemostasis, inflammation resolution, angiogenesis, growth, re‐epithelialisation and remodelling—phases that often overlap during healing.

The healing process primarily encompasses vascular contraction, haemostasis, collagen deposition and resolution of inflammation. MSCs have garnered considerable interest in regenerative medicine because of their capacity to secrete numerous factors that facilitate skin healing [32, 33, 34, 35, 36]. These cells can expedite re‐epithelialization, promote angiogenesis and directly differentiate into epithelial cells that express keratinocyte‐specific markers [37, 38]. In our study, we demonstrated that both A‐MSCs and UC‐MSCs aided in skin damage repair, albeit with differing effects. We identified significant differences in gene expression between UC‐MSCs and A‐MSCs through GO and KEGG analyses. Notably, upregulated genes in amniotic MSCs include *Tnip1*, *Cdh13*, *Laptm4b*, *Hipk2*, *Mmp1* and *Hbegf*. These genes are linked to various functions: for instance, for extracellular matrix organisation, *Mmp1* plays a vital role in ECM remodelling during wound healing [39, 40, 41]; for cell–cell adhesion, genes like *Cdh13* may enhance adhesion between amniotic MSCs and surrounding cells, promoting cellular aggregation and functional synergy to expedite healing [42, 43]; and for regulation of cell migration, upregulated genes related to cell migration could enhance the movement of fibroblasts and other repair‐related cells toward the wound site. Conversely, UC‐MSCs exhibited significant upregulation of genes such as *Bmper*, *Ptgs2*, *Fgf2*, *Timp3* and *Pdgfra*: for angiogenesis promotion; *Fgf2* is crucial for neovascularization during wound healing [44, 45, 46]; for anti‐inflammatory effects, *Ptgs2* is involved in regulating inflammatory responses [45, 47, 48]; and for extracellular matrix remodelling, *Timp3* regulates MMP activity to maintain a balance between collagen degradation and synthesis [49, 50, 51]. Overactive MMPs can degrade newly formed matrix and delay healing, while TIMP3 helps maintain ECM integrity by balancing proteolytic activity [52]. Additionally, TIMP3 has anti‐inflammatory properties and can inhibit angiogenesis under certain contexts, suggesting a complex role in fine‐tuning tissue remodelling. Increased TIMP3 expression in MSC‐treated wounds may reflect a controlled remodelling environment that prevents excessive matrix breakdown and scar formation. Tnip1 is known as a negative regulator of the NF‐κB signalling pathway, which plays a central role in inflammation and immune responses [53]. Tnip1 helps to suppress excessive inflammatory signalling, thereby preventing chronic inflammation and promoting resolution during wound healing. Moreover, elevated expression of Tnip1 in MSC‐treated wounds may contribute to a balanced inflammatory environment, reducing prolonged inflammation that otherwise impairs healing and leads to fibrosis or chronic wounds. Recent studies have also implicated Tnip1 in modulating macrophage polarisation toward an anti‐inflammatory M2 phenotype, which supports tissue regeneration and remodelling [54]. Based on these analyses, we conclude that A‐MSCs excel in tissue remodelling and cell migration capabilities owing to their upregulation of ECM organisation‐related genes. Conversely, UC‐MSCs are particularly effective in promoting angiogenesis and managing inflammatory responses through their secretions.

The application of MSCs in repairing skin injuries represents a promising strategy with significant potential; however, several challenges remain unresolved in clinical settings. Over the past 2–3 years, comparative studies of MSCs derived from different tissue sources—such as bone marrow, adipose tissue and umbilical cord—have provided valuable insights into their relative efficacy in wound healing [55]. UC‐MSCs exhibit superior proliferative and angiogenic potential compared to bone marrow‐derived MSCs, resulting in faster wound closure and enhanced neovascularization in diabetic wound models [56]. These findings highlight the importance of selecting the appropriate type of MSC based on specific treatment needs—whether to prioritise rapid healing or minimise scarring—is crucial. Additionally, factors such as viability of MSC cultures in vitro and their proliferative capacity can significantly affect treatment outcomes. Inflammatory microenvironment surrounding wounds also influences MSC functionality. In summary, understanding distinct properties of different MSC types can impact therapeutic choices in skin repair strategies. Ongoing research aimed at optimising these approaches is vital to improve clinical outcomes in wound healing processes. The present study is constrained to a single murine model, which may not fully recapitulate human wound healing dynamics. Furthermore, variability in MSC sources from donors and long‐term functional outcomes, such as scar formation, require further investigation. Future studies will integrate larger animal models and evaluate donor‐matched MSC batches to address these limitations.

## Author Contributions

**Nong‐er Shen:** investigation (equal), writing – original draft (equal). **Yue Wu:** data curation (equal), investigation (equal), project administration (equal). **Kaichuang Yang:** data curation (equal), investigation (equal), methodology (equal), project administration (equal). **Xiuling Xv:** data curation (equal), methodology (equal). **Gang Lu:** investigation (equal), methodology (equal). **Ruolang Pan:** conceptualization (equal), project administration (equal), writing – review and editing (equal). **Yang Jin:** conceptualization (equal), project administration (equal), writing – review and editing (equal).

## Ethics Statement

Tshe studies involving human participants were reviewed and approved by the ethics committee of S‐Evans Biosciences (no. 2020‐01). All experiments were conducted according to the protocols approved by the local Medical Animal Experiment Ethics Committee.

## Conflicts of Interest

The authors declare no conflicts of interest.

## Data Availability

The data that support the findings of this study are available from the corresponding author upon reasonable request.
